# Supplementary material for: A narrow range of transcript-error rates across the Tree of Life
Source: Sci Adv. 2025 Jul 11;11(28):eadv9898. doi: 10.1126/sciadv.adv9898 (PMC12248287; doi:10.1126/sciadv.adv9898)
Supplement: Supplementary file 1 — Supplementary Text Fig. S1 Legends for tables S1 to S10 References [file sciadv.adv9898_sm.pdf]

Science Advances
AAAS

Supplementary Materials for

A narrow range of transcript-error rates across the Tree of Life

Weiyi Li *et al.*

Corresponding author: Michael Lynch, mlynch11@asu.edu

Sci. Adv. **11**, eadv9898 (2025)
DOI: 10.1126/sciadv.adv9898

The PDF file includes:

Supplementary Text
Fig. S1
Legends for tables S1 to S10
References

Other Supplementary Material for this manuscript includes the following:

Tables S1 to S10

Supplementary Text

Theory for the Evolutionary Bounds on the Transcript-error Rate

Based on numerous direct observations on the effects of random amino-acid substitutions on protein performance (*62*, *62*, *68–72*), as well as results from numerous large-scale population-genetic analyses (*73–77*), we start with the assumption that the majority of errors in transcripts leading to nonsynonomous changes are deleterious upon translation. We wish then to evaluate the expected selective advantage of a genomic variant that improves transcriptional fidelity (or conversely the disadvantage of a variant that exacerbates the transcript-error rate), with the ultimate goal of ascertaining the degree to which such variants have substantial enough effects relative to the power of genetic drift to be perceived by selection. To achieve such an understanding, several factors must be considered: 1) the expected number of errors per transcript that manifest at the amino-acid sequence level; 2) the total steady-state number of transcripts/cell associated with each gene; and 3) the fitness effects of such errors.

As we assume that the fitness effects of transcript errors are revealed after translation, the following derivations will consider the rate of nonsynonymous errors per codon in mRNAs, u. Letting the error rate per nucleotide site be μ, because there are 3 nucleotide sites per codon, and $\sim 3/4$s of nucleotide substitutions cause an amino-acid substitution (as a consequence of the structure of the genetic code), $u \simeq 9\mu/4$. Letting L_i be the number of amino acids (codons) in a protein of type i, the numbers (j) of erroneous amino acids in individual protein molecules of this type will be approximately Poisson distributed with expectation uL_i,

$$P(j \mid uL_i) = \frac{e^{-uL_i}(uL_i)^j}{j!} \tag{S1}$$

Because transcript errors are generally singular events, and the mean number of protein molecules per active gene is generally $\gg 1$, individual variant proteins within a cell will generally be just a fraction of the total pool of molecules for specific genetic loci. This raises the question of the degree to which the fitness effects of single transcript errors are manifest at the cellular level. As with variant alleles at a genetic locus, transcription and translation errors might behave in an additive, recessive, or dominant fashion, with the magnitude of the latter two conditions depending on the number of transcripts per cell. Thus, there is a need to know the fraction of the pool of proteins present in a cell at any one time that are derived from a particular transcript (which might contain a unique transcript error).

Letting $\tilde{n}_t$ be the steady-state number of transcripts per cell for a particular gene, the total number of transcripts produced per cell cycle is

$$n_t = \tilde{n}_t \left(1 + \frac{d_t T}{\ln(2)}\right) \tag{S2a}$$

where d_t is the transcript decay rate, and T is the cell-division time. Likewise, the total number of proteins produced per cell cycle is

$$n_p = \tilde{n}_p \left(1 + \frac{d_p T}{\ln(2)}\right) \tag{S2b}$$

It then follows that the fraction of proteins in the cell associated with one particular transcript at any time is approximately

$$\frac{1}{\tilde{n}_t}\left(\frac{\ln(2) + d_p T}{\ln(2) + d_t T}\right) = \frac{k}{\tilde{n}_t} \tag{S3}$$

The fact that decay rates of transcripts are generally larger than those for proteins leads to the expectation that $k < 1$, although rough estimates can only be obtained for a few organisms. For *E. coli* and a few other bacteria, $d_t \simeq 10$ /hour (*78–81*), whereas average $d_p \simeq 0.2$ /hour for several bacteria (*82*, *83*), so assuming $T \simeq 0.4$ hours for a bacterial cell growing at maximum rate, $k \simeq 0.16$ Similarly, for the yeast *S. cerevisiae*, $d_t \simeq 5$ /hour (*84*, *85*), $d_p \simeq 1.4$ /hour (*86*), and assuming $T \simeq 3$ hours at maximum growth rate, $k \simeq 0.32$. Finally, for mouse fibroblast cells, $d_t \simeq 0.1$ /hour and $d_p \simeq 0.02$ /hour (*87*), which under the assumption of $T \simeq 24$ hours at maximum growth rate leads to $k \simeq 0.38$ These limited observations suggest that k may be relatively constant across species, in which case its exact value will be irrelevant to the scaling relationships determined below, and it will be ignored for the time being, assuming relative constancy across species and genes.

Thus, given that the average fraction of protein molecules for a particular locus associated with individual transcripts is inversely proportional to the average transcript number, letting $\tilde{n}_{t,i}$ be the number of steady-state transcripts per cell for protein i, a flexible function that allows for alternative modes of dilution of effects is

$$f(\tilde{n}_{t,i}) = \frac{1}{\tilde{n}_{t,i}^x} \tag{S4}$$

which equals 1.0 when $\tilde{n}_{t,i} = 1$ (effects are fully felt), and converges to 0.0 (effects are completely masked) as $\tilde{n}_{t,i} \to \infty$ at a rate that depends on the exponent x. When $x = 1, f(\tilde{n}_{t,i}) = 1/\tilde{n}_{t,i}$ and the number of copies of a protein has no effect, as the number of error-containing proteins, which is proportional to $\tilde{n}_{t,i}$, is compensated by the dilution effect, i.e., $\tilde{n}_{t,i} \cdot f(\tilde{n}_{t,i}) = 1$. Values of $0 < x < 1$result in a relatively slow decline in the dilution effect with increasing $\tilde{n}_{t,i}$ (with $x = 0$ implying complete dominance), i.e., synergism of errors, whereas $x > 1$ results in a relatively rapid decline in $f(\tilde{n}_{t,i})$ (i.e., increasingly recessive effects of errors). More complicated two-parameter models that allow for non-log-linear behavior might be warranted, but direct empirical observations will be required to reveal the utility of such nuances.

For each locus i, the expected fractional reduction in fitness associated with the transcript-error burden (s_i) will depend on the number of transcripts per cell over which the errors are distributed $\tilde{n}_{t,i}$, the degree of expression of individual errors $f(\tilde{n}_{t,i})$, the distribution of the numbers of errors per protein $P(j \mid uL_i)$, and the average reduction in fitness associated with fully expressed deleterious mutations. From the references cited above, it is known that the latter is generally $<$ 0.1, and based on the transcript-error rates reported herein and typical gene lengths, the number of errors per protein will generally be $\ll 10$. Thus, letting δ be the fitness loss per single error in a single transcript if fully revealed, and assuming independent effects of individual errors, the total reduction in fitness resulting from a protein containing j errors is $(1-\delta)^j \simeq 1 - e^{-j\delta}$. It then follows that

$$s_i = \tilde{n}_{t,i} \cdot f(\tilde{n}_{t,i}) \cdot \sum_{j=1}^{L_i} P(j \mid uL_i) \cdot \left(1 - e^{-j\delta_i}\right) \tag{S5a}$$

$$\simeq \tilde{n}_{t,i} \cdot f(\tilde{n}_{t,i}) \cdot \left[1 - \exp\left(-\frac{uL_i\delta_i}{1+\delta_i}\right)\right] \tag{S5b}$$

$$\simeq \tilde{n}_{t,i} \cdot f(\tilde{n}_{t,i}) \cdot (uL_i) \cdot \delta_i \tag{S5c}$$

where the latter approximation assumes $\delta_i \ll 1$.

Further noting that the error loads at each target locus will be $\ll 1$, and assuming independent effects across loci, relative to a baseline fitness of 1.0 for an error-free individual, the selective disadvantage of a genotype with error rate u is

$$s(u) \simeq 1 - \exp\left(-\sum_{i=1}^{P} s_i\right) \tag{S6}$$

where P is the number of loci in the proteome. Recalling Equation S5c, the selection differential between two variants with transcript-error rates u and $(1+\Delta)u$ is then

$$s(\Delta) \simeq \sum_{i=1}^{P} \tilde{n}_{t,i} \cdot f(\tilde{n}_{t,i}) \cdot \Delta \cdot u \cdot L_i \cdot \delta_i \tag{S7}$$

where transformation to the additive scale follows from the fact that the terms in the exponent are $\ll 1$. Further simplification is possible if it is assumed that the copy number effects, $\tilde{n}_{t,i}. f(\tilde{n}_{t,i})$, are independent of the lengths of loci, L_i, and error effects, δ_i,

$$s(\Delta) \simeq [\bar{n} \cdot \phi_n] \cdot [\Delta \cdot u \cdot P\bar{L}] \cdot \bar{\delta} \tag{S8}$$

where overlines denote mean values, and $\phi_n = \{1 - [x(1-x)C_n^2/2]\}/\bar{n}^x$, obtained by Taylor-series expansion of $\tilde{n}_{t,i} \cdot f(\tilde{n}_{t,i})$, is the average dilution factor, with $\bar{n}$ and C_n denoting the mean and coefficient of variation in expression level.

Equation S8 shows that the genome-wide fitness consequences of transcript errors are a function of three quantities: 1) the net influence of the steady-state numbers of transcripts per gene and the degree of dilution of error effects (the first bracketed expression); 2) the total error rate per expressed proteome, $uP\bar{L}$; and 3) the average effect of an undiluted amino-acid substitution, $\bar{\delta}$, which must be equivalent to the average homozygous effect of a genomic mutation. Our ultimate goal is to define the drift barrier to the evolution of transcript fidelity (μ^*), i.e., the error rate at which the next incremental improvement $s(\Delta) \simeq 1/N_e$,

$$\mu^* \propto \frac{1}{\Delta \cdot N_e \cdot \bar{n}\phi_n \cdot P\bar{L} \cdot \bar{\delta}} \tag{S9}$$

The granularity of mutational changes in alleles influencing the error rate (Δ) operates as a simple scaling factor, but does not change the form of the scaling relationship with respect to other cellular features -- the higher the value of Δ, the greater the difference of allelic effects, and hence the greater the efficiency of selection for a lower error rate.

These results show that the scaling properties of the drift barrier depend on both the population-genetic environment (N_e) and the cellular environment (the remaining terms in Equation S9). As these two sets of features are not necessarily independent, a strict inverse relationship between the evolved error rate and N_e may not hold. To gain further insight into these mutual dependencies, we consider the ways in which some of the terms in Equation S9 scale with organism size. From Lynch and Trickovic 2020 (*5*),

$$N_e \simeq (8 \times 10^7)V^{-0.18}, \tag{S10}$$

where a conversion from dry weight (in the original paper) to organism volume (V in μm^3) has been made using a universal relationship of dry weight (ng) $= 0.00057V^{0.92}$ (Lynch 2024 (*40*)). From Lynch and Marinov 2015 (*42*),

$$\bar{n} \simeq 3V^{0.28} \tag{S11}$$

and for the dilution-factor effect, $C_n = 2$ can be applied as a first-order approximation (based on an absence of phylogenetic pattern C_n and an observed range of 1 to 3).

Supplementary Figure

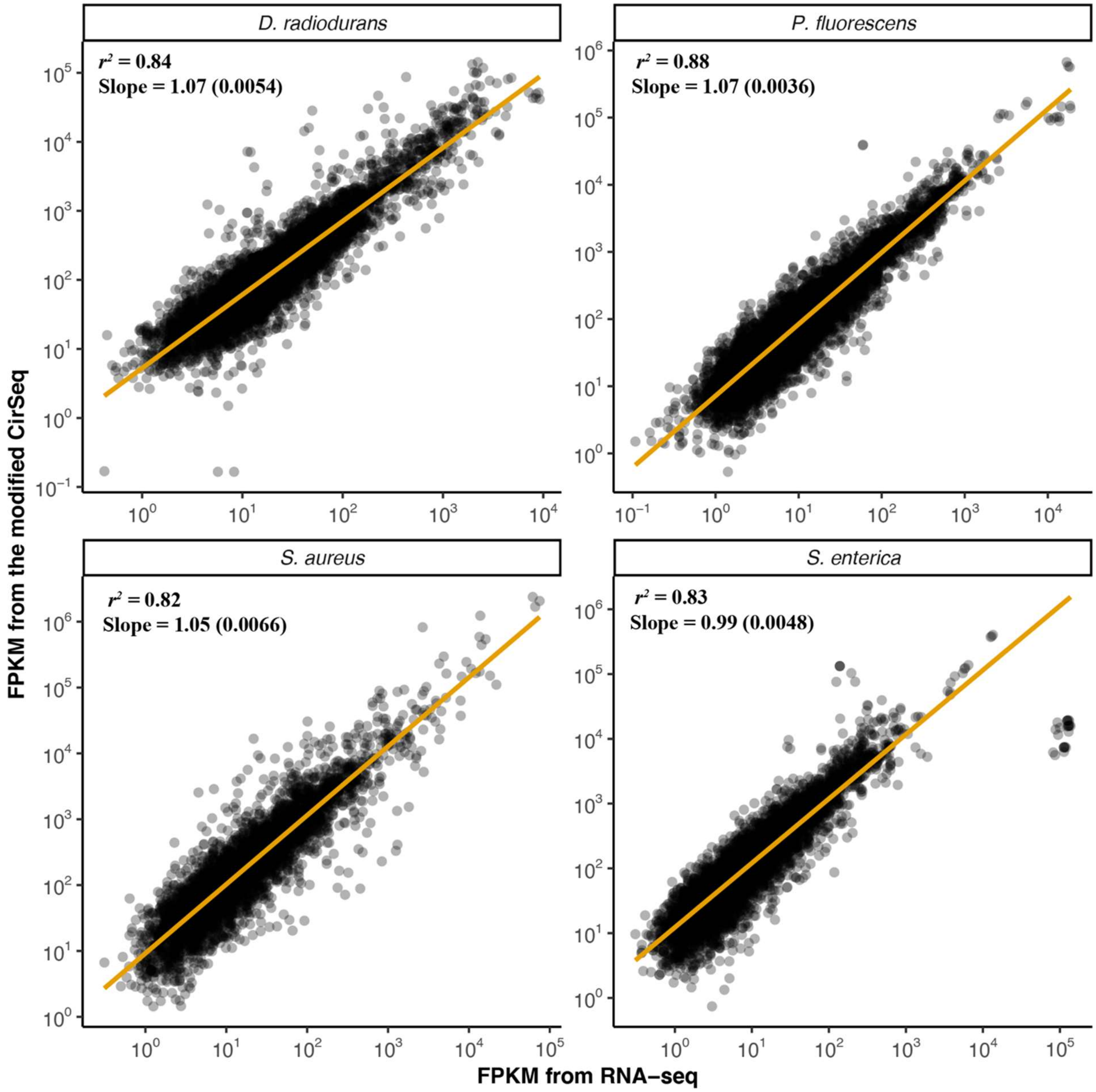

Figure S1. Least-square regressions of gene expression levels estimated from regular RNA-seq and modified CirSeq. Constructions of the modified CirSeq library requires a circularization procedure of RNA molecules that might result in a bias in estimates of expression level of different genes. To evaluate this issue, we prepared regular RNA-seq and modified CirSeq libraries in parallel for four species. FPKM (Fragments Per Kilobase of transcript per Million mapped reads) calculated from two types of libraries are positively correlated, indicating procedures of preparing the modified CirSeq library do not introduce biases in abundances of RNA fragments derived from different genes. Standard errors of the regression coefficients are given in parentheses.

Supplementary Tables

Table S1. Summary of results, estimates, and citations used in analysis and hypothesis testing.

Table S2. Prokaryote strains, culture media, and growth conditions.

Table S3. Estimates of effective population sizes for prokaryotic species.

Table S4. Details of regression analyses of transcript-error rates vs. expression levels for protein-coding genes. Expression levels of genes (FPKM) were obtained from CirSeq reads, which provide estimates for expression levels consistent with regular RNA-seq reads (Figure S1). In Method 1, the generalized linear model from Meer et al. 2020 was adapted (Materials and Methods). This Poisson distribution-based model was applied to individual protein-coding genes to evaluate potential correlations between expression levels and transcript-error rates. P-values of the slope were obtained by performing ANOVA tests with the χ^2 option in R to evaluate whether including the slope significantly improves the generalized linear model. In Method 2, least-squares linear regression analyses were carried out on transcript-error rates and log-transformed expression levels of individual protein-coding genes. In Method 3, the only difference from Method 2 is that expression levels of individual genes were not log-transformed, i.e., the least-squares linear regressions were carried out on arithmetic-arithmetic scales. Breusch-Pagan tests were used to test for homoscedasticity in linear-regression models generated by Method 2 and 3 (Table S5). In Method 4, logistic regression analyses were performed on transcript-error rates and expression levels of individual protein-coding genes on arithmetic-arithmetic scales. Regressions with P-values $< 0.05/21$ (Bonferroni correction for regression analyses for 21 species) are considered statistically significant. Significant positive and negative regressions are highlighted in blue and red respectively.

Table S5. Checking homoscedasticity in linear-regression models using the Breusch-Pagan test. Homoscedasticity of linear-regression model fits relating transcript-error rates with gene-expression levels was assessed using Breusch-Pagan tests. Two scales were used for constructing linear-regression models. On the logarithmic-arithmetic scale (Method 2 in Table S4), gene-expression levels were log-transformed before constructing linear-regression models. On the arithmetic-arithmetic scale (Method 3 in Table. S4), gene-expression levels and transcript-error rates were used directly for regression models.

Table S6. Hotspot genes for transcript errors detected in each species.

Table S7. Protein-coding genes used to estimate transcript-error rates of chloroplast-encoded RNAPs.

Table S8. The molecular spectra of transcript errors from nuclear/nucleoid chromosomal mRNAs of each replicate.

Table S9. Outlier loci for transcript errors excluded from each species.

Table S10. Evaluation of different consensus read coverage thresholds for detecting transcript errors.

REFERENCES AND NOTES

1. T. Ohta, Slightly deleterious mutant substitutions in evolution. *Nature* **246**, 96–98 (1973).

2. D. L. Hartl, D. E. Dykhuizen, A. M. Dean, Limits of adaptation: The evolution of selective neutrality. *Genetics* **111**, 655–674 (1985).

3. M. Lynch, *The Origins of Genome Architecture* (Oxford Univ. Press, 2007).

4. M. Lynch, M. S. Ackerman, J.-F. Gout, H. Long, W. Sung, W. K. Thomas, P. L. Foster, Genetic drift, selection and the evolution of the mutation rate. *Nat. Rev. Genet.* **17**, 704–714 (2016).

5. M. Lynch, B. Trickovic, A theoretical framework for evolutionary cell biology. *J. Mol. Biol.* **432**, 1861–1879 (2020).

6. M. Lynch, F. Ali, T. Lin, Y. Wang, J. Ni, H. Long, The divergence of mutation rates and spectra across the Tree of Life. *EMBO Rep.* **24**, e57561 (2023).

7. M. Lynch, The cellular, developmental and population-genetic determinants of mutation-rate evolution. *Genetics* **180**, 933–943 (2008).

8. M. Lynch, Evolution of the mutation rate. *Trends Genet.* **26**, 345–352 (2010).

9. M. Lynch, The lower bound to the evolution of mutation rates. *Genome Biol. Evol.* **3**, 1107–1118 (2011).

10. D. I. Lou, J. A. Hussmann, R. M. McBee, A. Acevedo, R. Andino, W. H. Press, S. L. Sawyer, High-throughput DNA sequencing errors are reduced by orders of magnitude using circle sequencing. *Proc. Natl. Acad. Sci. U.S.A.* **110**, 19872–19877 (2013).

11. A. Acevedo, R. Andino, Library preparation for highly accurate population sequencing of RNA viruses. *Nat. Protoc.* **9**, 1760–1769 (2014).

12. J.-F. Gout, W. Li, C. Fritsch, A. Li, S. Haroon, L. Singh, D. Hua, H. Fazelinia, Z. Smith, S. Seeholzer, K. Thomas, M. Lynch, M. Vermulst, The landscape of transcription errors in eukaryotic cells. *Sci. Adv.* **3**, e1701484 (2017).

13. W. Li, M. Lynch, Universally high transcript error rates in bacteria. *eLife* **9**, e54898 (2020).

14. C. Fritsch, J.-F. Gout, S. Haroon, A. Towheed, C. Chung, J. LaGosh, E. McGann, X. Zhang, Y. Song, S. Simpson, P. S. Danthi, B. A. Benayoun, D. Wallace, K. Thomas, M. Lynch, M. Vermulst, Genome-wide surveillance of transcription errors in response to genotoxic stress. *Proc. Natl. Acad. Sci. U.S.A.* **118**, e2004077118 (2021).

15. C. Chung, B. M. Verheijen, X. Zhang, B. Huang, A. Coakley, E. McGann, E. Wade, O. Dinep-Schneider, J. LaGosh, M.-E. Anagnostou, S. Simpson, K. Thomas, M. Ernst, A. Rattray, M. Lynch, M. Kashlev, B. A. Benayoun, Z. Li, J. Strathern, J.-F. Gout, M. Vermulst, The fidelity of transcription in human cells. *Proc. Natl. Acad. Sci. U.S.A.* **120**, e2210038120 (2023).

16. B. S. Masters, L. L. Stohl, D. A. Clayton, Yeast mitochondrial RNA polymerase is homologous to those encoded by bacteriophages T3 and T7. *Cell* **51**, 89–99 (1987).

17. G. M. Cheetham, T. A. Steitz, Structure of a transcribing T7 RNA polymerase initiation complex. *Science* **286**, 2305–2309 (1999).

18. M. Gaspari, N.-G. Larsson, C. M. Gustafsson, The transcription machinery in mammalian mitochondria. *Biochim. Biophys. Acta* **1659**, 148–152 (2004).

19. T. Börner, A. Y. Aleynikova, Y. O. Zubo, V. V. Kusnetsov, Chloroplast RNA polymerases: Role in chloroplast biogenesis. *Biochim. Biophys. Acta* **1847**, 761–769 (2015).

20. J.-F. Gout, W. K. Thomas, Z. Smith, K. Okamoto, M. Lynch, Large-scale detection of in vivo transcription errors. *Proc. Natl. Acad. Sci. U.S.A.* **110**, 18584–18589 (2013).

21. C. C. Traverse, H. Ochman, Conserved rates and patterns of transcription errors across bacterial growth states and lifestyles. *Proc. Natl. Acad. Sci. U.S.A.* **113**, 3311–3316 (2016).

22. K. S. Reid-Bayliss, L. A. Loeb, Accurate RNA consensus sequencing for high-fidelity detection of transcriptional mutagenesis-induced epimutations. *Proc. Natl. Acad. Sci. U.S.A.* **114**, 9415–9420 (2017).

23. K. M. Meer, P. G. Nelson, K. Xiong, J. Masel, High transcriptional error rates vary as a function of gene expression level. *Genome Biol. Evol.* **12**, 3754–3761 (2020).

24. J. P. DeLong, J. G. Okie, M. E. Moses, R. M. Sibly, J. H. Brown, Shifts in metabolic scaling, production, and efficiency across major evolutionary transitions of life. *Proc. Natl. Acad. Sci. U.S.A.* **107**, 12941–12945 (2010).

25. M. Lynch, B. Trickovic, C. P. Kempes, Evolutionary scaling of maximum growth rate with organism size. *Sci. Rep.* **12**, 22586 (2022).

26. N. Alic, N. Ayoub, E. Landrieux, E. Favry, P. Baudouin-Cornu, M. Riva, C. Carles, Selectivity and proofreading both contribute significantly to the fidelity of RNA polymerase III transcription. *Proc. Natl. Acad. Sci. U.S.A.* **104**, 10400–10405 (2007).

27. J. F. Sydow, P. Cramer, RNA polymerase fidelity and transcriptional proofreading. *Curr. Opin. Struct. Biol.* **19**, 732–739 (2009).

28. M. Graille, B. Séraphin, Surveillance pathways rescuing eukaryotic ribosomes lost in translation. *Nat. Rev. Mol. Cell Biol.* **13**, 727–735 (2012).

29. S. Kervestin, A. Jacobson, NMD: A multifaceted response to premature translational termination. *Nat. Rev. Mol. Cell Biol.* **13**, 700–712 (2012).

30. I. Golding, E. C. Cox, RNA dynamics in live *Escherichia coli* cells. *Proc. Natl. Acad. Sci. U.S.A.* **101**, 11310–11315 (2004).

31. S. Proshkin, A. R. Rahmouni, A. Mironov, E. Nudler, Cooperation between translating ribosomes and RNA polymerase in transcription elongation. *Science* **328**, 504–508 (2010).

32. M. H. Larson, J. Zhou, C. D. Kaplan, M. Palangat, R. D. Kornberg, R. Landick, S. M. Block, Trigger loop dynamics mediate the balance between the transcriptional fidelity and speed of RNA polymerase II. *Proc. Natl. Acad. Sci. U.S.A.* **109**, 6555–6560 (2012).

33. P. Eser, L. Wachutka, K. C. Maier, C. Demel, M. Boroni, S. Iyer, P. Cramer, J. Gagneur, Determinants of RNA metabolism in the *Schizosaccharomyces pombe* genome. *Mol. Syst. Biol.* **12**, 857 (2016).

34. A. Lisica, C. Engel, M. Jahnel, É. Roldán, E. A. Galburt, P. Cramer, S. W. Grill, Mechanisms of backtrack recovery by RNA polymerases I and II. *Proc. Natl. Acad. Sci. U.S.A.* **113**, 2946–2951 (2016).

35. S. Ucuncuoglu, K. L. Engel, P. K. Purohit, D. D. Dunlap, D. A. Schneider, L. Finzi, Direct characterization of transcription elongation by RNA polymerase I. *PLOS ONE* **11**, e0159527 (2016).

36. M. B. Ardehali, J. T. Lis, Tracking rates of transcription and splicing in vivo. *Nat. Struct. Mol. Biol.* **16**, 1123–1124 (2009).

37. K. T. Hiriyanna, T. Ramakrishnan, Deoxyribonucleic acid replication time in Mycobacterium tuberculosis H37 Rv. *Arch. Microbiol.* **144**, 105–109 (1986).

38. B. Stillman, Cell cycle control of DNA replication. *Science* **274**, 1659–1663 (1996).

39. H. Myllykallio, P. Lopez, P. López-García, R. Heilig, W. Saurin, Y. Zivanovic, H. Philippe, P. Forterre, Bacterial mode of replication with eukaryotic-like machinery in a hyperthermophilic archaeon. *Science* **288**, 2212–2215 (2000).

40. M. R. Lynch, *Evolutionary Cell Biology: The Origins of Cellular Architecture* (Oxford Univ. Press, 2024).

41. R. Carter, G. Drouin, The increase in the number of subunits in eukaryotic RNA polymerase III relative to RNA polymerase II is due to the permanent recruitment of general transcription factors. *Mol. Biol. Evol.* **27**, 1035–1043 (2010).

42. M. Lynch, G. K. Marinov, The bioenergetic costs of a gene. *Proc. Natl. Acad. Sci. U.S.A.* **112**, 15690–15695 (2015).

43. D. A. Drummond, C. O. Wilke, The evolutionary consequences of erroneous protein synthesis. *Nat. Rev. Genet.* **10**, 715–724 (2009).

44. D. M. McCandlish, J. B. Plotkin, Transcriptional errors and the drift barrier. *Proc. Natl. Acad. Sci. U.S.A.* **113**, 3136–3138 (2016).

45. M. Ehrenberg, C. G. Kurland, Costs of accuracy determined by a maximal growth rate constraint. *Q. Rev. Biophys.* **17**, 45–82 (1984).

46. C. G. Kurland, M. Ehrenberg, Optimization of translation accuracy. *Prog. Nucleic Acid Res. Mol. Biol.* **31**, 191–219 (1984).

47. C. G. Kurland, M. Ehrenberg, Growth-optimizing accuracy of gene expression. *Annu. Rev. Biophys. Biophys. Chem.* **16**, 291–317 (1987).

48. S. W. Peltz, A. B. Hammell, Y. Cui, J. Yasenchak, L. Puljanowski, J. D. Dinman, Ribosomal protein L3 mutants alter translational fidelity and promote rapid loss of the yeast killer virus. *Mol. Cell. Biol.* **19**, 384–391 (1999).

49. V. Pezo, D. Metzgar, T. L. Hendrickson, W. F. Waas, S. Hazebrouck, V. Döring, P. Marlière, P. Schimmel, V. De Crécy-Lagard, Artificially ambiguous genetic code confers growth yield advantage. *Proc. Natl. Acad. Sci. U.S.A.* **101**, 8593–8597 (2004).

50. J. M. Bacher, W. F. Waas, D. Metzgar, V. de Crécy-Lagard, P. Schimmel, Genetic code ambiguity confers a selective advantage on *Acinetobacter baylyi*. *J. Bacteriol.* **189**, 6494–6496 (2007).

51. T. Pan, Adaptive translation as a mechanism of stress response and adaptation. *Annu. Rev. Genet.* **47**, 121–137 (2013).

52. L. Ribas de Pouplana, M. A. S. Santos, J.-H. Zhu, P. J. Farabaugh, B. Javid, Protein mistranslation: Friend or foe? *Trends Biochem. Sci.* **39**, 355–362 (2014).

53. M. A. Santos, C. Cheesman, V. Costa, P. Moradas-Ferreira, M. F. Tuite, Selective advantages created by codon ambiguity allowed for the evolution of an alternative genetic code in *Candida* spp. *Mol. Microbiol.* **31**, 937–947 (1999).

54. X. Wang, T. Pan, Stress response and adaptation mediated by amino acid misincorporation during protein synthesis. *Adv. Nutr.* **7**, 773S–779S (2016).

55. P. Leeds, J. M. Wood, B. S. Lee, M. R. Culbertson, Gene products that promote mRNA turnover in *Saccharomyces cerevisiae*. *Mol. Cell. Biol.* **12**, 2165–2177 (1992).

56. J. N. Dahlseid, J. Puziss, R. L. Shirley, A. L. Atkin, P. Hieter, M. R. Culbertson, Accumulation of mRNA coding for the ctf13p kinetochore subunit of *Saccharomyces cerevisiae* depends on the same factors that promote rapid decay of nonsense mRNAs. *Genetics* **150**, 1019–1035 (1998).

57. J. T. Mendell, S. M. Medghalchi, R. G. Lake, E. N. Noensie, H. C. Dietz, Novel Upf2p orthologues suggest a functional link between translation initiation and nonsense surveillance complexes. *Mol. Cell. Biol.* **20**, 8944–8957 (2000).

58. J. Hodgkin, A. Papp, R. Pulak, V. Ambros, P. Anderson, A new kind of informational suppression in the nematode *Caenorhabditis elegans*. *Genetics* **123**, 301–313 (1989).

59. S. M. Medghalchi, P. A. Frischmeyer, J. T. Mendell, A. G. Kelly, A. M. Lawler, H. C. Dietz, Rent1, a trans-effector of nonsense-mediated mRNA decay, is essential for mammalian embryonic viability. *Hum. Mol. Genet.* **10**, 99–105 (2001).

60. M. Vermulst, A. S. Denney, M. J. Lang, C.-W. Hung, S. Moore, M. A. Moseley, J. W. Thompson, V. Madden, J. Gauer, K. J. Wolfe, D. W. Summers, J. Schleit, G. L. Sutphin, S. Haroon, A. Holczbauer, J. Caine, J. Jorgenson, D. Cyr, M. Kaeberlein, J. N. Strathern, M. C. Duncan, D. A. Erie, Transcription errors induce proteotoxic stress and shorten cellular lifespan. *Nat. Commun.* **6**, 8065 (2015).

61. W. Sung, M. S. Ackerman, S. F. Miller, T. G. Doak, M. Lynch, Drift-barrier hypothesis and mutation-rate evolution. *Proc. Natl. Acad. Sci. U.S.A.* **109**, 18488–18492 (2012).

62. M. Lynch, K. Hagner, Evolutionary meandering of intermolecular interactions along the drift barrier. *Proc. Natl. Acad. Sci. U.S.A.* **112**, E30–38 (2015).

63. S. A. Frank, Robustness and complexity. *Cell Syst.* **14**, 1015–1020 (2023).

64. D. Kim, G. Pertea, C. Trapnell, H. Pimentel, R. Kelley, S. L. Salzberg, TopHat2: Accurate alignment of transcriptomes in the presence of insertions, deletions and gene fusions. *Genome Biol.* **14**, R36 (2013).

65. C. Trapnell, D. G. Hendrickson, M. Sauvageau, L. Goff, J. L. Rinn, L. Pachter, Differential analysis of gene regulation at transcript resolution with RNA-seq. *Nat. Biotechnol.* **31**, 46–53 (2013).

66. S. Wang, H. Luo, Dating the bacterial Tree of Life based on ancient symbiosis. *Syst. Biol.*, **syae071** (2025).

67. H. Long, W. Sung, S. Kucukyildirim, E. Williams, S. F. Miller, W. Guo, C. Patterson, C. Gregory, C. Strauss, C. Stone, C. Berne, D. Kysela, W. R. Shoemaker, M. E. Muscarella, H. Luo, J. T. Lennon, Y. V. Brun, M. Lynch, Evolutionary determinants of genome-wide nucleotide composition. *Nat. Ecol. Evol.* **2**, 237–240 (2018).

68. P. D. Keightley, A. Eyre-Walker, Joint inference of the distribution of fitness effects of deleterious mutations and population demography based on nucleotide polymorphism frequencies. *Genetics* **177**, 2251–2261 (2007).

69. C. D. Huber, B. Y. Kim, C. D. Marsden, K. E. Lohmueller, Determining the factors driving selective effects of new nonsynonymous mutations. *Proc. Natl. Acad. Sci. U.S.A.* **114**, 4465–4470 (2017).

70. B. Y. Kim, C. D. Huber, K. E. Lohmueller, Inference of the distribution of selection coefficients for new nonsynonymous mutations using large samples. *Genetics* **206**, 345–361 (2017).

71. M. Lynch, R. Gutenkunst, M. Ackerman, K. Spitze, Z. Ye, T. Maruki, Z. Jia, Population genomics of *Daphnia pulex*. *Genetics* **206**, 315–332 (2017).

72. T. R. Booker, P. D. Keightley, Understanding the factors that shape patterns of nucleotide diversity in the house mouse genome. *Mol. Biol. Evol.* **35**, 2971–2988 (2018).

73. D. D. Axe, Extreme functional sensitivity to conservative amino acid changes on enzyme exteriors. *J. Mol. Biol.* **301**, 585–595 (2000).

74. H. H. Guo, J. Choe, L. A. Loeb, Protein tolerance to random amino acid change. *Proc. Natl. Acad. Sci. U.S.A.* **101**, 9205–9210 (2004).

75. P. A. Lind, O. G. Berg, D. I. Andersson, Mutational robustness of ribosomal protein genes. *Science* **330**, 825–827 (2010).

76. B. P. Roscoe, K. M. Thayer, K. B. Zeldovich, D. Fushman, D. N. A. Bolon, Analyses of the effects of all ubiquitin point mutants on yeast growth rate. *J. Mol. Biol.* **425**, 1363–1377 (2013).

77. E. Firnberg, J. W. Labonte, J. J. Gray, M. Ostermeier, A comprehensive, high-resolution map of a gene's fitness landscape. *Mol. Biol. Evol.* **31**, 1581–1592 (2014).

78. J. A. Bernstein, A. B. Khodursky, P.-H. Lin, S. Lin-Chao, S. N. Cohen, Global analysis of mRNA decay and abundance in *Escherichia coli* at single-gene resolution using two-color fluorescent DNA microarrays. *Proc. Natl. Acad. Sci. U.S.A.* **99**, 9697–9702 (2002).

79. G. Hambraeus, C. von Wachenfeldt, L. Hederstedt, Genome-wide survey of mRNA half-lives in *Bacillus subtilis* identifies extremely stable mRNAs. *Mol. Genet. Genomics* **269**, 706–714 (2003).

80. Y. Taniguchi, P. J. Choi, G.-W. Li, H. Chen, M. Babu, J. Hearn, A. Emili, X. S. Xie, Quantifying *E. coli* proteome and transcriptome with single-molecule sensitivity in single cells. *Science* **329**, 533–538 (2010).

81. C. Dressaire, F. Picard, E. Redon, P. Loubière, I. Queinnec, L. Girbal, M. Cocaign-Bousquet, Role of mRNA stability during bacterial adaptation. *PLOS ONE* **8**, e59059 (2013).

82. P.-J. Lahtvee, A. Seiman, L. Arike, K. Adamberg, R. Vilu, Protein turnover forms one of the highest maintenance costs in *Lactococcus lactis*. *Microbiology* **160**, 1501–1512 (2014).

83. C. Trötschel, S. P. Albaum, A. Poetsch, Proteome turnover in bacteria: Current status for *Corynebacterium glutamicum* and related bacteria. *J. Microbial. Biotechnol.* **6**, 708–719 (2013).

84. Y. Wang, C. L. Liu, J. D. Storey, R. J. Tibshirani, D. Herschlag, P. O. Brown, Precision and functional specificity in mRNA decay. *Proc. Natl. Acad. Sci. U.S.A.* **99**, 5860–5865 (2002).

85. B. Neymotin, R. Athanasiadou, D. Gresham, Determination of in vivo RNA kinetics using RATE-seq. *RNA* **20**, 1645–1652 (2014).

86. A. Belle, A. Tanay, L. Bitincka, R. Shamir, E. K. O'Shea, Quantification of protein half-lives in the budding yeast proteome. *Proc. Natl. Acad. Sci. U.S.A.* **103**, 13004–13009 (2006).

87. B. Schwanhäusser, D. Busse, N. Li, G. Dittmar, J. Schuchhardt, J. Wolf, W. Chen, M. Selbach, Global quantification of mammalian gene expression control. *Nature* **473**, 337–342 (2011).
